# ‘I’m not going to leave someone to die’: carriage of naloxone by police in Scotland within a public health framework: a qualitative study of acceptability and experiences

**DOI:** 10.1186/s12954-023-00750-9

**Published:** 2023-02-19

**Authors:** Elizabeth M. Speakman, Peter Hillen, Inga Heyman, Jennifer Murray, Nadine Dougall, Elizabeth V. Aston, Andrew McAuley

**Affiliations:** 1 School of Medicine, University of Dundee, Dundee, UK; 2 School of Health and Social Care, Edinburgh Napier University, Edinburgh, UK; 3 School of Health and Life Sciences, Glasgow Caledonian University, Glasgow, UK

**Keywords:** Naloxone, Police, Drug deaths, Public health, Harm reduction

## Abstract

**Background:**

Scotland has one of the highest rates of drug-related deaths (DRDs) per capita in Europe, the majority of which involve opioids. Naloxone is a medication used to reverse opioid-related overdoses. In efforts to tackle escalating DRDs in many countries, naloxone is increasingly being provided to people who are likely first responders in overdose situations. This includes non-healthcare professionals, such as police officers. A pilot exercise to test the carriage and administration of naloxone by police officers was conducted in selected areas of Scotland between March and October 2021. The aim of the study was to explore the acceptability and experiences of naloxone carriage and administration by police in Scotland.

**Methods:**

The study comprised of two stages. Stage 1 involved in-depth one-to-one qualitative interviews with 19 community stakeholders (people with lived experience, family members, support workers). Stage 2 involved a mixture of in-depth one-to-one interviews and focus groups with 41 police officers. Data were analysed thematically, and the findings from the two stages were triangulated to develop overarching themes and subthemes.

**Results:**

By the end of the pilot, 808 police officers had been trained in the use of intranasal naloxone. Voluntary uptake of naloxone kits among police officers who completed training was 81%. There were 51 naloxone administration incidents recorded by police officers at suspected opioid-related overdose incidents during the pilot. Most officers shared positive experiences of naloxone administration. Naloxone as a first aid tool suited their role as first responders and their duty and desire to preserve life. Perceived barriers included concerns about police undertaking health-related work, potential legal liabilities and stigmatising attitudes. The majority of participants (and all community stakeholders) were supportive of the pilot and for it to be expanded across Scotland.

**Conclusions:**

Police carriage of naloxone is an acceptable and potentially valuable harm reduction tool to help tackle the DRDs crisis in Scotland. However, it requires appropriate integration with existing health and social care systems. The intervention lies at the intersection between public health and policing and implies a more explicit public health approach to policing.

## Introduction

The ongoing crisis of opioid-related drug deaths represents a significant global public health challenge [1], causing significant mortality and morbidity [2]. To address this, a multi-agency collaborative response adopting a public health approach is required [3]. More recent work has highlighted both the benefits and necessity of police collaboration with health and social care partners and, when working with people living with complex needs or vulnerabilities, adopting a public health approach [4, 5].

In 2020 there were 1339 drug-related deaths (DRDs) registered in Scotland [6], a rate 3.5 times higher than the UK average and the highest in Europe [6]. The total number of DRDs in Scotland has increased substantially over the last 20 years with 4.6 times as many deaths in 2020 when compared with the year 2000. Although more than one substance was found to be present in the body in 93% of people who suffered DRDs, the most prevalent drugs detected were opioids such as heroin/morphine and methadone which were present in 1,192 DRDs in 2020 (89% of the total) [6].

Naloxone is a medication with a long history of use as a first aid measure to reverse the effect of an opioid-related overdose [7]. It is medically indicated for use as an emergency antidote to opioid-related overdoses and reverses the suppression of the respiratory system. Naloxone may be administered by intravenous or intramuscular injection or by spraying into a person’s nostril (intranasal). Naloxone causes no harm if given to an individual who is mistakenly believed to have taken opioids, and does not cause dependence[8]. There is a growing evidence base for the positive impact of THN on reducing drug deaths [9, 10]. A recent systematic review from Australia refuted misconceptions that THN would lead to increased substance use, finding instead that it ‘has a net benefit in terms of drug use behaviours’ [11].

In the UK, naloxone has been legally authorised for use by members of the public in an emergency since 2005 [12] and in 2015 naloxone became available without prescription [13]. In 2011, the Scottish Government implemented the world’s first National Naloxone Programme (NNP), providing Take-Home-Naloxone (THN) kits to people who use drugs likely to witness an overdose [14]. Over time, the NNP programme has expanded to allow supplies of naloxone to be made available to friends, family and professionals likely to witness an overdose.

In the USA, naloxone’s combination of safety and effectiveness prompted expansion of supplies to first responders who were not healthcare professionals [15]. As the opioid crisis worsened in the USA [16], some police forces, for example in Ohio, Indiana and Pennsylvania, have implemented the carriage of naloxone among officers in an attempt to reduce deaths [16–18]. Literature on US police attitudes discusses positive views and receptiveness to naloxone training [17, 19], as well as evidence of stigma and negativity from frontline law enforcement officers [20].

Following the US example, more countries have begun issuing naloxone to police forces, including Canada [21] and Australia [22]. In the UK, a ‘Nasal Naloxone Pilot’ was run by West Midlands Police during 2019–2020 [23], which concluded by recommending widening naloxone access to police officers across the region. Naloxone is now being introduced in various police forces throughout the UK.

Police Scotland (PS) was established in 2013 as a single police service for Scotland, with the stated purpose of policing to enhance the safety and wellbeing of individuals and communities enshrined in legislation [24]. More recently, PS has expressed strategic commitment to taking a public health approach to policing through collaboration with Public Health Scotland, committing to principles of safety, protection, resilience and wellbeing [25]. In 2020, Police Scotland developed a drugs strategy that aims to reduce harm and develop a public health approach [26]. In response to the year-on-year increase in DRDs, and the recommendation of the Scottish Drugs Deaths Taskforce, PS conducted a pilot to test the training, carriage and administration of intranasal naloxone by police officers who might encounter suspected overdoses in the course of their duties [27]. The pilot was conducted between March and October 2021 in selected areas in Scotland. This paper reports primarily on qualitative data collected from police officers and community participants, drawn from an independent mixed methods evaluation of the pilot [28].

## Methods

### Design

The study aimed to explore the acceptability and experiences of naloxone carriage and administration by police in Scotland, as considered by police and people in the community. This was achieved in two stages. Stage 1: In-depth one-to-one semi-structured qualitative interviews were carried out with community stakeholders (people with lived or living experiences, family members of people with lived experience and support workers from specialist third sector agencies). Stage 2: In-depth one-to-one semi-structured interviews and focus groups were carried out with police officers. The study was part of a larger mixed-methods process evaluation of a pilot project conducted by PS on police carriage and administration of naloxone in Scotland [28]. The pilot project involved officers being trained in the use of intranasal naloxone in three geographical pilot areas in Scotland (Glasgow East, Dundee, Falkirk). The training was subsequently rolled out to police officers in Caithness, to custody officers in Glasgow and Falkirk and to community police officers in Stirling during the course of the pilot.^1^ The pilot period was between 1 March and 31 October 2021 and the evaluation was conducted between 1 March 2021 and 17 February 2022. This paper discusses the qualitative data from the evaluation to allow in-depth of exploration of the findings. Background data on the uptake and administration of naloxone were supplied by PS. ‘Uptake’ refers to officers voluntarily taking a naloxone kit at the end of the training course for carriage as part of their equipment.

### Participants

Stage 1: Nineteen community stakeholders were recruited to take part in one-on-one interviews. This included people with lived or living experience of opioid use (LEOU) (*N* = 9, family members of people with LEOU *N* = 4) and third sector staff with experience of supporting people with LEOU (*N* = 10). Some participants had lived, family and support staff experience, hence the total numbers of role-experiences exceeding the total number of participants. The community stakeholders had a range of experience with drug overdose and naloxone, including personal experience of overdosing, observing people overdosing, observing naloxone being administered, administering naloxone themselves and/or being trained in the administration of naloxone. Participants were based in Glasgow (*N* = 5), Falkirk (*N* = 2), Dundee (*N* = 4), Argyll and Bute (*N* = 1), Aberdeenshire (*N* = 3), Aberdeen (1), Scottish Borders (*N* = 1), Edinburgh (*N* = 1) and Scotland-wide (*N* = 1). Similar numbers of males and females took part and a range of age groups were represented. Participants were recruited purposefully via email communication from either their place of employment or via support organisations that they were in contact with. Participants received the project information sheet, privacy notice and a consent form within the email. Anyone wishing to take part clicked on a digital *Novi Survey* consent link embedded within the email, where they provided their consent and contact details, and were followed up by a member of the research team to arrange a suitable time for the interview. Participants who were not in paid employment at the time of interview received a £20 gift voucher post-participation. Participants had the option of withdrawing their data up to two weeks post-participation; no one requested this.

Stage 2: Forty-one police officers took part in the study in total. The majority were male (*N* = 30, 73%) and aged between 25 and 44 years (*N* = 32, 78%). Eighteen police officers were recruited to take part in one-to-one interviews, and an additional 23 officers took part in one of four focus groups. All participants had taken part in the PS naloxone training as part of the pilot and responded to a study recruitment email sent to all trained officers post-training. Participants self-selected to take part via a dedicated *Novi Survey* link within the email; thereafter, they were contacted by a member of the research team. Participants could withdraw their data up to 2 weeks post-participation; no one chose to do this. Five of the participants had been involved in the development and/or implementation of the pilot naloxone training programme. Officers were based in Glasgow (*N* = 14), Dundee (*N* = 9), Falkirk (*N* = 10), Caithness (*N* = 3), and five officers were ‘Scotland wide’ (*N* = 5; i.e. involved in nationwide operations, rather than being attached to any particular division). Thirty-four had experience of attending to an overdose, 26 had seen naloxone administered, and 13 had administered naloxone. Additional participant demographics are shown in Table 1. Although officer gender was recorded, the data sample was not large enough to indicate whether this was a factor in officer attitudes.

**Table 1:** Demographics of police officer participants

Participants *N* (%)	
**Age group (years)**	
< 25	2 (4.88)
25–34	19 (46.34)
35–44	13 (31.71)
45–44	7 (17.01)
**Years’ experience within Police Scotland**	
1–5	17 (41.46)
6–10	8 (19.51)
11–15	5 (12.20)
16–20	4 (9.76)
21–25	3 (7.32)
> 26	4 (9.76)
**Rank**	
Constable	29 (70.73)
Sergeant	8 (19.51)
Inspector	1 (2.44)
Chief Inspector or Higher	3 (7.32)

### Materials

Standardised information sheets, consent forms, privacy notices and debriefing information sheets were provided to all participants via email. Standardised topic guides were used to facilitate semi-structured conversations within the interviews and focus groups. Encrypted recording devices were used to audio-record the interviews and focus groups; interviews were conducted using telephone or *MS Teams* software.

### Procedure

Stage 1: Interviews with community stakeholders were conducted either by telephone or online video call. Interviews were conducted by ES or JM and lasted an average of 33 min.

Stage 2: Interviews with police officers were conducted by ES in person, by telephone or by online video call and lasted an average of 42 min. The four police officer focus groups were conducted in person by IH, PH and ES and lasted an average of 86 min.

Interviews and focus groups were supported through the use of a topic guide to facilitate semi-structured conversations. The data were audio-recorded, transcribed verbatim and anonymised prior to analysis.

### Ethical permissions

Ethical approval was provided by Edinburgh Napier University’s School of Health and Social Care’s Research Integrity Committee. Permission to conduct the research was further obtained from PS and where individuals from third sector organisations were interviewed, permission was also supplied by their organisation.

### Analyses

Data were analysed separately for the two stages; for both stages the analysis process was the same. A thematic analysis approach, broadly following Braun and Clarke’s (2006) guidance, was followed, and *NVIVO 20* software was used to support the analysis through organisation of themes and sub-themes throughout the process [29]. Themes were initially drawn using the topic guides and research objectives to guide this. However, throughout the process effort was made to identify new and emergent themes from the data. Data were categorised into initial sub-themes. These were grouped and re-grouped and were then organised under key themes. After data for Stages 1 and 2 were analysed separately using NVivo 20, the two datasets were combined, synthesised together, then re-grouped into the final themes. Data were cross-checked and analysed by at least two members of the research team (ES, PH, IH, JM), before the whole research team worked together to consolidate the findings.

## Results

### Naloxone training and uptake

Naloxone training was conducted in PS offices around Glasgow, Dundee, Falkirk, Stirling and Caithness between 1 March and 31 October 2021. Training was compulsory for officers in the pilot areas although the decision whether to carry naloxone following training was voluntary. Those who volunteered to take part were given a personal issue pouch containing two intranasal naloxone (Nyxoid) packs. A total of 808 police officers took part in the naloxone training, 87% of the eligible workforce in the pilot areas and 5% of all Police Scotland officers. Of the 808 officers trained, 656 (81%) chose to take naloxone packs.

### Naloxone administration incidents

There were 51 recorded naloxone administration incidents by police officers during the pilot. Individuals received on average between one and two doses of naloxone from police officers who administered it. Police records indicate that all the recipients of naloxone administration by police survived these overdose events and no adverse effects were reported. Each of the incidents happened under one of four circumstances: a response to an emergency call (26), attending a general incident (11), on general duty (9) or being flagged down by a member of the public (5). The majority of incidents occurred in public spaces (i.e. on the street) (27) or in a private residence (12), a communal space (e.g. on a bus, in a doorway) (9), in a police vehicle (1), a police office (1) or in custody (1). After police officers administered naloxone, the Scottish Ambulance Service (SAS) attended 45 incidents: 41 were taken to hospital, while four refused further treatment, left the scene or were left in care of third party. In the other six cases, recipients refused further treatment (4) or were conveyed to hospital by police officers (2).

### Experience of naloxone administration

The majority of police participants (34 of 41) had experience of attending an overdose. Of these, 26 had observed naloxone being administered (in most cases by ambulance personnel) and 13 had personal experience of administering naloxone themselves. Those who had observed naloxone being administered witnessed its effectiveness in reviving individuals who had overdosed on opioids. One potential challenge that was mentioned on several occasions, was that the recipient could respond aggressively. However, this was not given as a reason not to administer naloxone.

*‘I’d seen Naloxone being administered by paramedics, and it’s amazing when you’ve actually seen if it you’ve actually seen it in person. They go from pretty much unconscious to completely awake. The downside to that sometimes is people get a bit angry with you because you’ve ruined their hit a lot of the time.’ (PC01)*

Officers who had personally administered intranasal naloxone were overwhelmingly positive about its usefulness and effectiveness in reversing the effects of an opioid overdose. The following account is one example.

*‘There was an unconscious male outside one of the local supermarkets and they were looking for some assistance. The ambulance wasn’t nearby so I went along to see if I could be of any assistance ... it appeared that he was under the influence of opioids so I phoned the ambulance to let them know that I was there and that I had naloxone and I was going to administer it. I administered one dose and he didn’t really come round straight away so I was a bit sceptical as to if it would actually work or not, and after kind of ten minutes or so he came round. It was quite amazing to see actually how it worked.’ (PC02)*

Officers were positive about the safety of using intranasal naloxone as there was no risk of needle stick injury. They had some reservations about having to carry another piece of equipment on their person, but this was not perceived to be a major obstacle. Some officers were aware that naloxone was of limited benefit where people had overdosed on a range of substances (poly drug use). This limitation was also expressed by a number of community participants, for example, one of the support staff emphasised that whilst naloxone continues to be important as opioids are present in most drug-related deaths, naloxone cannot reverse the effects of all overdose incidents and therefore there is still more to be done.

### Organisational mistrust and legal concerns

The pilot was controversial, with the main staff association, the Scottish Police Federation (SPF) questioning its benefits. While they had a number of objections, a key point of contention was ‘legal risk’ [30]. The SPF said they would not support officers in the event of an investigation or legal claim following an administration of naloxone where the recipient comes to harm. PS assured officers that they should not be concerned about any legal investigation if a person were to come to harm following an administration of naloxone. The Crown Office had confirmed that there would be no prosecution in such a situation and the Head of Investigations for the Police Investigations and Review Commissioner (PIRC) stated in training that there would be no PIRC investigation. Nevertheless, this conflict between PS and SPF exposed a degree of organisational mistrust from police officers and doubt around legal support from either organisation.

*‘The biggest barrier for me at the very start was the Federation turned round and saying they don’t support it and all that, and they’re not going to be there.’ (PC-FG1)*

*‘I think basically police officers have zero confidence in the Federation or the organisation they work for as a whole to back them up if something like that was to happen.’ (PC12)*

However, many officers were not worried about legal repercussions from administering naloxone in an emergency and believed that the legal concerns of other officers are unjustified.

*‘If we’ve got the assurance there that, you know, you can’t overdose on it, there’s no lasting effects, and that we’re not going to be held responsible for anyone if there are ill effects of it then that’s good enough for me… So as long as you used it in accordance with your training, then I was quite happy with the assurances given by the [Chief Constable].’ (PC18)* *‘We have a big metal stick that we can use…and guns and tasers and stuff like that, but nobody has an issue with doing that. So why do we have an issue? The liability with that is a lot greater than sticking a spray up somebody’s nose.’ (PC08)*

### Police role

Many police officers argued that a police officer’s duty to preserve life was paramount and naloxone was an opportunity to proactively achieve this. This duty was underlined by a recognition that police officers were frequently first responders to overdose incidents, either in response to emergency calls or being flagged down in the street by members of the public. As such, officers felt that it was appropriate that they should be able to provide emergency first aid, including naloxone, until ambulance support arrives. For many officers this duty and opportunity to save a life overcame any other reservations or concerns.

*‘At the end of the day the police are there to save people’s lives, as strange as it sounds. So I’m not going to stand around and watch someone die.’ (PC10)*

*‘Our core duty is the preservation of life and keeping people safe. If the organisation says you’re going to carry something to assist in the preservation of life, it’s hard to argue against that.’ (PC17)*

Some officers expressed concern that the carriage of naloxone was another step towards police officers taking on an ‘ever-increasing’ medical role and taking on workload that should be the remit of paramedics.

*‘I just think where does it stop? Do we then start carrying tourniquets and all that sort of stuff in case we…because…we do go to violent incidents, we go to stabbings and things like that, so where does it stop to what we are dealing with as first aiders?’ (PC-FG2)*

This was usually described as ‘mission creep’. The perception of an expanding first aid role was contextualised in broader concerns about workload, particularly in response to increasingly having to support people with mental health needs. However, for most officers, concerns about the changing role of police and about workload were overridden by the duty to preserve life. One police interviewee had no problem with this public health role, pointing to the historical roots of the focus on looking after people, including the welfare of residents, unsanitary living conditions and the hygiene of the streets.

All community participants who were interviewed, believed that the intervention was suitable for police officers as first responders and that it fitted well with the police’s duty to save lives.

*‘It’s about preserving life isn't it? It’s not about, wait a minute I’ll just check what my role is... I don't see it as a job. I see it as saving life and about support for somebody in the community if you had to and I don't understand why you would not do it.’ Female, LEOU (LDE08)*

Several community participants were sympathetic with the burden of workload and stress that police face, for example, one member of support staff highlighted the stressful nature of the incidents police have to deal with and their need for more support. I

However, they saw that using naloxone could only support officers in their role. The alternative could be dealing with a person’s death.

### Partnership working with ambulance services

The Scottish Ambulance Service (SAS) are the primary responders to drug overdoses in Scotland. PS works to support SAS when a suspected overdose occurs, as they are often first on the scene. SAS is called to respond to all overdose incidents, irrespective of whether a police officer is able to administer naloxone. Central to police concerns was how their naloxone capability was to be integrated with the response from ambulance services. Some police respondents welcomed the opportunity to provide greater support for ambulance services, others felt it was inappropriate. Despite some officers reporting prompt ambulance responses, others stated that they had experienced ambulance delays and sometimes poor communication between ambulance call handlers and attending police officers, including a lack of awareness of the pilot from ambulance personnel. Many officers said this was a long-standing problem of insufficient ambulance resources and that it increased police workload, with the police being unfairly required to fill gaps in emergency services. The following quotes illustrate contrasting experiences.

*‘Any kind of incidents where we’ve administered naloxone the ambulances have been really quite prompt at coming.’ (PC02)*

*‘I think there is a lack of communication generally between ourselves and ambulance, and I think it does lead to us not being prioritised where we should be prioritised. We’ve had unconscious males lying in the street without an ambulance for, you know, an hour, but they’re not responding to us.’ (PC-FG3)*

Several respondents made the point that concerns about slow ambulance response could in fact support the case for greater police access to naloxone. Officers in rural areas recognised the benefit of providing support to other emergency services.

*‘The response time for both ourselves and the other emergency services can be quite vast... you just muck in and everybody does everything here, and it is accepted that an ambulance might take a long time because it is remote and rural, so you would do everything you could.’ (PC04)*

### Follow-up support

All participants agreed that naloxone could only be part of the solution and an integrated approach was necessary, with support from all services, if the drugs crisis was to be tackled effectively. Officers recognised the life-saving capacity of naloxone, and that while it may only have a small impact on DRDs nationally, it provides an opportunity to link people who use drugs into support services.

*‘Naloxone isn’t going to cure anything, I don’t think… but it might be just the thing that keeps somebody alive and gives them an opportunity for somebody to intervene to stop them becoming another casualty next week.’ (PC18)*

*‘There’s people that come to our attention for overdoses who we’ve never met before, and genuinely they’ve never had any help because nobody knew they even existed. In those cases, you can keep them going until the paramedics come and then people, like at the hospital, the psychiatrist, actually, can get involved because they can see that these people are in crisis. It buys people a second chance.’ (PC13)*

Some officers felt that follow-up support was lacking, with increased risk of repeated overdoses, and death after administration.

*‘It’s like fighting a fire because we’re administering naloxone but the services aren’t coming in behind us to back-fill that.’ (PC-FG2)*

*‘I think obviously this is to stop people dying from it, it doesn’t resolve the whole problem... we’re solving the problem of people dying but we’re not solving the problem of the drugs issue that we have.’ (PC10)*

Also, some officers believed (incorrectly) that after administering naloxone they would be required to stay with the individual until the effect of drugs had worn off (if the individual refused to go to hospital—or alternatively, if the ambulance refused to take the individual). There was a strong perception that this would increase police workload or, if they did not stay with the individual, risk a Police Investigations and Review Commissioner (PIRC) enquiry if the person subsequently came to harm. Some officers were concerned that there was a lack of training and ‘safe spaces’ to support vulnerable drug users following an overdose.

Several community participants noted that individuals who had overdosed and were revived were at risk of repeated overdose and perhaps repeated administrations of naloxone. This was not seen as an argument against naloxone, but rather pointed to the need for support following near fatal overdoses.

*‘We should be looking at prevention and intervention at every opportunity, absolutely every opportunity. It doesn’t matter what service, you know, preventing drug-related death is a societal responsibility. It’s not the addiction services. It’s not criminal justice. Every single person, every human, in society and the community, has got a responsibility to try and prevent death.’ Female support staff (SM02)*

Several police officers shared the view that policing could not tackle drug issues alone, but in emphasising the need for effective partnership working, some officers highlighted the police role as enforcement and others emphasised a public health approach.

*‘There’s criminality associated with [the drugs issue], but I think it’s primarily a public health issue that we need to get to the root causes of... why are people taking it in the first place?... I think it’s definitely a public health issue, and a real partnership approach.’ (PC18)*

### Attitude to people who use drugs

Police officers demonstrated a range of attitudes toward people who used illicit drugs. Many officers showed compassion and concern and some talked of having been affected by problematic substance use personally, including through family and close friends.

*‘You end up getting to know certain people and then you understand what they have went through, even at a young age... To be honest, it is a shame, certainly they are trying their best and they just fall back and take drugs again.’ (PC07)*

*‘When they die it has an impact on us... we deal with these people from when, you know, they’re young teenagers and after 12 years you see them going on to be adults, and then to see them dead and have to deal with their death is traumatic. It’s horrible. You miss them.’ (PC13)*

Some accounts indicated underlying stigma, perhaps through holding discriminatory attitudes, or due to having a lack of understanding about problematic drug use. For example, several officers considered problematic drug use to be a ‘lifestyle choice’ and it was reported that the term ‘junkie’ is a descriptor used by some officers.^2^

*‘Some of the responses from cops is, you know, why do I want to save a junkie? You’re only saying that because you view them as criminals, not as people.’ (PC-FG3)*

The following quote from a police officer recounting an administration of naloxone, suggests that they held a stigmatising view towards the individual.

*‘I can only describe his behaviour as deplorable. He was abusive to us and horrible to the ambulance staff who had just saved his life, no gratitude whatsoever, going mental at them because they had ripped his shirt... once they did leave we were like that, that’s who we’re saving, somebody who’s just so ungrateful and probably away to offend again, to get his next hit and then cause more misery because he’s not a nice individual.’ (PC15)*

Community participants shared a range of contrasting views around the attitudes of police officers towards people who use drugs. Several participants shared positive accounts of police officers who were proactive and compassionate in their support of people who use drugs. Some participants believed that police officer attitudes towards people who use drugs have improved over time, and that younger officers, in particular, had more positive attitudes. Community officers were also considered to have greater understanding than front-line response officers. For example, one member of support staff commented that police have less stigmatising attitudes and are more understanding now being trauma-informed and another focused on communication:

*‘A real mixed bag of experience with the police…I think over the last two or three years Police Scotland have made I think quite significant shifts into being a more caring organisation ... So there is certainly, I think, from my experience been quite a significant shift over the last couple of years and I've probably got some really good examples where police have been really positive, really flexible, wanting to work together, pretty happy to share information with good levels of communication, so that's certainly been my experience in the last wee while.’ Male support staff (SM07).*

## Discussion

The overall findings of this evaluation strongly supported the implementation of naloxone carriage and administration across PS. At the end of training, 81% of officers who underwent training voluntarily chose to carry naloxone packs. While the study was focused on processes rather than outcomes, the 51 naloxone administration incidents by police officers in the pilot areas demonstrated the potential of the intervention for police. The number of administrations equated to almost 8% of total packs carried by officers. This was comparable to usage of 9% recorded for take-home naloxone in the community [32]. The accounts of officers who had administered naloxone as a first aid intervention highlighted the ability of officers and the acceptability of the intervention. All 19 community participants were strongly supportive of the pilot and its roll out across Scotland.

The research highlighted factors that may facilitate police officers’ adoption of naloxone: they are often first responders to overdoses and may be in a position to reverse an overdose until ambulance services arrive; naloxone provides an effective first aid tool which allows police to carry out their duty to preserve life; many police officers care about the communities they work in and want to have a positive impact on the lives of vulnerable drug users; police officers are in a good position to work in partnership with emergency services to support people who overdose; and also to collaborate with other health and social care services to facilitate support following a near fatal overdose. All participants agreed that intranasal naloxone increased ease of use and therefore acceptability. Although community participants thought the risk of an aggressive response could be a barrier, most officers said it would not deter them from naloxone administration.

The research findings also highlighted challenges to the acceptability of police carriage and administration of naloxone. Opposition from the SPF created a discourse of risk around the intervention and indicated a culture of distrust both in PS and the SPF [30]. The discourse of risk revolved around two primary concerns: anxiety about legal repercussions if a person came to harm after an administration of naloxone by a police officer; and the perception that police are taking on a more medicalised role that might result in police doing work that should be done by paramedics.

Naloxone is a licensed medication in the UK, regulated by the National Institute for Clinical Excellence (NICE). It can be supplied to members of the public without prescription. SPF’s portrayal of naloxone administration as a risky behaviour which could lead to investigation and prosecution is contradicted by a large evidence base that it is exceptionally safe [8] and has been used to good effect by police forces across the world for many years: in the USA since at least 2014 [33–36] and in the UK since 2019 [23]. Side effects are rare and are far outweighed by its lifesaving benefit [37]. Furthermore, intranasal naloxone is easy to administer and removes any risk of needlestick injury.

Although police officers are frequently first responders to an emergency, including suspected overdoses, they are under no statutory legal obligation to administer naloxone or any other type of first aid because they are not healthcare professionals. This is instead left to the discretion of the attending police officer. Both PIRC and the Crown Office have stated that they will not be investigating or prosecuting police officers for administering naloxone in cases where a person has subsequently come to harm. These assurances are in relation both to deaths in police custody and deaths following police contact. No record could be found of any legal claim (successful or otherwise) having been brought against any first responder who administered naloxone in an emergency, either in the UK or elsewhere.

Concerns about legal liability may seem unjustified based on the evidence, but the SPF arguments were raised repeatedly by officers unwilling to carry naloxone for this reason. As the concerns remain pervasive among police officers, an effective response is required to facilitate clarity and quell anxieties. The need for clarity around legislation has been reflected in Canadian research [21]. Since the completion of this evaluation, PS has provided reassurances about the legality of administering naloxone appropriately and the protections if a person were to die following an administration, stating that ‘naloxone in no way differs to any other first aid or other action taken by officers to try and save a person’s life’ and that ‘should a person suffer a fatal overdose, the presence, or not, of naloxone will have no bearing on whether or not the matter is treated as a death following police contact.’ All police officers on duty are protected by PS’s Legal Assistance Programme [38]. Despite this, representatives of the SPF remain resistant to the initiative.

The second key concern and challenge to acceptability related to police role and workload. While the police recognised the considerable pressure on health services, many felt that they were in a similar position, and being unfairly required to meet shortfalls in other services. Many felt under-supported with increasing demand to respond to mental health emergencies, rather than on fighting crime. Mental health and drug use are interlinked and require a response from all services [39]. The lack of support both in response and follow-up was cited as a source of frustration for some police officers. Equipping officers with naloxone gives them the capacity to save a life rather than having to deal with the death of an individual. In practice, this should reduce workload rather than increase it. However, officers still need to be supported through partnership working to deal with the ongoing burdens of supporting people who overdose. Pike reported similar responses from officers in Kentucky where officers expressed frustration at police time and resources being used to respond to overdose-related calls and provide emergency medical care [40].

Most police officers were fully aware that DRDs were a major public health problem in Scotland and that health services in Scotland were under considerable strain, particularly within the context of the COVID-19 pandemic. The majority of officers understood the importance of supporting ambulance services, and they saw naloxone as an invaluable first aid tool that police could administer before paramedics arrived. Officers in rural areas in particular saw the value of being able to provide first aid in locations where ambulance delay is more common. While there was goodwill towards ambulance services and a desire to help, this was combined with frustration.

Ambulance delays came up repeatedly as a concern of police officers, although some interviewees reported prompt responses to calls. Some accounts of ambulance delay and miscommunication were from personal experience; others were reported second-hand. These accounts differed from official policy as represented by the SAS. Data on ambulance response times indicated some delays and inconsistency in responses, but COVID-19 undoubtedly affected response times [28]. Other anecdotal data suggest that police were facing extra demands due to pressure on ambulance services [41].

Participants in police and community groups agreed that naloxone could only be one part of the response to the DRDs crisis. Critical to the success of this initiative is integrated partnership working with health and social care services. Equipping police officers with naloxone should be seen as just one aspect of a comprehensive multi-agency response to the DRDs crisis in Scotland, or a whole systems response [25]. This point was made by Lowder et al. in response to the opioid epidemic in the USA. They declared that ‘use of a harm-reduction tool like naloxone provision is only a single component of a larger community-based response to the opioid epidemic’ [39]. Lowder et al. argue that naloxone needs to be complemented by buy-in from criminal justice stakeholders and community treatment providers to enable diversion from acute setting (jail and hospital). This compares with recent research by Antoniou et al. which found that THN on its own is not enough to reduce DRD rates, and that a multi-faceted approach with access to additional harm reduction, treatment and other interventions is required [42].

Scotland has already made progress in terms of diversion of people in possession of Class A drugs in certain circumstances [43]. Police have a key role in diversion, as set out in Scotland’s drug policy:

Diverting those with problematic alcohol and drug use away from the justice system and into treatment support, and other interventions that reduce harm and preserve life, is essential. This approach needs to run through how the police lead the work to control the supply of drugs, sentencing, the provision of treatment and support in prison setting, as well as supporting continuity of care on release [44].

PS has developed a range of follow-up initiatives including the ‘Non-Fatal Overdose Pathway’ in Dundee and the ‘Positive Outcomes Project’ in Glasgow. These projects facilitate follow-up with people who have experienced a near fatal overdose through collaboration with specialist third sector agencies. However, the research indicated that follow-up initiatives were not consistent across Scotland. Developing a robust multiagency response to overdoses will require clear processes and minimum standards for follow up initiatives across Scotland. Further research and ongoing evaluation is required to assess the effectiveness of follow-up initiatives.

It is crucial that police engagement with people who use substances does not treat problem drug use as an isolated issue [45, 46]. Several of the police officers interviewed for this project identified the relationship between problem drug use, broader mental health issues and social disadvantage. PS has made some headway in taking an integrated approach towards problem drug use through Custody Hubs which recognize that people with problematic drug use are likely to have a range of needs, such as mental health problems and homelessness, which cannot be met by treatment services alone [44]. If it is acknowledged that problem drug use may often be one factor within a range of multiple complex needs, partnership initiatives ought to have a broad scope. This may mean developing partnership working between police officers and mental health specialists. Alternatively, it may mean the provision of a ‘safe space’ for people who are under the influence of drugs to be supported, something strongly recommended by police participants and supported by Gaeta et al. [47].

Many police officers demonstrated compassion towards people who use drugs, and this was recognised by community participants. There were encouraging examples of PS and individual officers working more effectively with support services in recent years, with more emphasis on care and support, than law enforcement. Both community participants and some police officers mentioned that community officers seemed to have a greater understanding than front-line response officers. Many community participants believed that naloxone carriage would improve relations between the police and people who use drugs. However, police and community participants reported experiences of stigma from some officers. Additionally, some of the language used by officers in the research indicated stigmatising attitudes towards drugs users and a lack of knowledge about problem drug use. It could also be argued that officers who opposed the initiative without a robust argument for doing so, were influenced by stigmatising attitudes within policing culture. It was argued by some community participants that ongoing resistance from the SPF was evidence of stigma, since they do not oppose, or require legal reassurances, for police use of other first aid tools, such as a defibrillator.

Community participants recommended that officers should receive more in-depth training to develop understandings of problem drug use and to address stigma. While stigma is present in public attitudes in Scotland [48], police officers may have a particular tendency to stigmatise people who use drugs, and also be opposed to harm reduction strategies [21, 49]. Developing a stigma training course in PS was indicated as a particular objective in the Scottish drugs strategy, ‘Rights, Respect and Recovery’ [44] and the results of this evaluation lend support to this. PS has since committed to provide stigma training to officers after one year of service. Ongoing evaluation of police stigma towards drug users and the effectiveness of training will be important as naloxone carriage is rolled out across PS, as research suggests that increased exposure to drug overdoses may increase stigmatising views [14].

It is clear that Scotland is suffering a worsening public health crisis of DRDs [6]. Emergency services, including police forces, are a valuable resource in facing this challenge, although this role is also controversial. Police officers are in the unique position of having a duty both to uphold the law and also to protect public safety—duties which may appear contradictory when faced with a person who has taken illegal drugs. This tension between enforcement and harm reduction was identified by Punch and James who observed that establishing cooperation between these two worlds ‘has been a prolonged and vacillating process’ [50].

In Scotland, the Police and Fire Reform (Scotland) Act 2012 specifies an obligation on Scottish police officers ‘to protect life’ (Section 20 (c)) and ‘to improve the safety and well-being of persons, localities and communities in Scotland’ (Section 32 (a)). Therefore, the duty to protect life is a priority responsibility for the police. Yet while the police role in protecting public safety is unarguable, it has not always been obvious that ‘public safety’ necessarily includes public health approaches to harm reduction. Nevertheless, the specifically public health role of the police was visible during the Covid-19 pandemic when the police were responsible for enforcing stay-at-home orders and were called to significantly increased numbers of mental health incidents [51, 52]. Adding naloxone to the first aid tools which officers are already accustomed to makes their public health role more explicit.

The predominant positive aspect identified in interviews and focus groups with police officers is that a police officer’s duty to preserve life is paramount and naloxone will save lives. This fundamental point outweighed any remaining concerns which some officers might have about ambulance delay or legal liability (although many who supported naloxone did not have these concerns). For these officers, naloxone (like any effective first aid tool), creates both a duty and an opportunity to save a life. This could be both as a first responder in literally saving the person from imminent death and also in subsequently referring the person to drug support or treatment services.

Following the publication of our research [28], the immediate impact was that the Chief Constable mandated all PS officers would be issued with naloxone as an emergency treatment for overdoses [53]. This was a promising step towards PS embracing a public health approach to policing, a *responsibility* that has been advocated in research by White et al. [54]:

[Police] officers’ acceptance of this public health responsibility and their willingness to administer naloxone are critical prerequisites to an effective response to the opioid crisis… Police officer acceptance of this role will save lives. Officers are frequently the first on scene of an opioid overdose, and time is critical. Life or death can hinge on a matter of seconds (p. 8).

PS has expressed strategic commitment to taking a public health approach, committing to principles of safety, protection, resilience and wellbeing for the ‘people, places and communities in Scotland’ [25]. Our research has highlighted the potential benefits of police carrying naloxone, but also clarified that more needs to be done, in partnership with other services, to reduce DRDs, support vulnerable people who use drugs and tackle the harms associated with problematic drug use. Progress will require the development of a ‘public health ethic’ in both policing strategy and practice as described by Del Pozo et al. [55]:

‘Police discretion guided by a public health ethic takes the profession’s putative role of protecting life and delivering public safety and operationalizes it with decisions that equitably improve health outcomes.’

Del Pozo et al. [55], drawing on Tyndall and Dodd [56], argue that an ethical response to drug use and overdose must provide a strong social support system, address stigma and discrimination, improve access to treatment and promote harm reduction interventions. These points strongly align with our research findings especially in terms of supporting the carriage of naloxone, improving partnership working with emergency services, addressing stigma through training and developing follow-up partnerships. A whole system approach is required, through ‘applying systems thinking, methods and practice to better understand public health challenges and identify collective actions’ [57].

## Conclusion

Scotland is facing an unprecedented number of DRDs that are at record levels. Since opioids are implicated in the vast majority of DRDs, naloxone is an essential intervention for saving lives [6]. Naloxone is an evidence-based, safe, first aid intervention that has been promoted by the Scottish Government for over a decade [58]. Police officers are often first responders to drug overdoses [58]. They are therefore in a position to offer first aid to people who may be overdosing before ambulance services can attend. Administering naloxone in a timely fashion could help save a person’s life.

Our research findings indicated that a majority of police officers who participated held a positive view of the carriage and administration of naloxone by police. The high uptake of intranasal naloxone kits by officers who attended the training (81%) presents a general indication of the acceptability of the intervention. The main concerns regarding naloxone relayed by police officers were around potential for increased workload, anxiety around the changing role of policing and unsubstantiated fears of legal repercussions if a person was harmed. Community participants overwhelmingly supported the pilot and saw no reason why it should not be standard practice for police officers to carry naloxone.

The research findings presented here demonstrate the feasibility and appropriateness of intranasal naloxone as a first aid tool for police officers. This harm reduction intervention should be adopted within a wider public health approach to policing, employing a whole systems approach [25, 46, 55, 59]. Police responses (such as carriage and administration of naloxone) to health problems must be delivered within the context of a strong and well-resourced multi-agency partnership approach. The fact that the ambulance service continued to be the primary responder to overdoses during the pilot in Scotland was important. The police service cannot and should not replace professional healthcare services, and greater integration with, and input from other services is required, e.g. via follow-up pathways. Further research is required to determine the efficacy of partnership working between police, ambulance and other services to determine how this could be improved to better support people at risk of overdose. In particular, follow-up initiatives need to be evaluated. The presence of stigmatising attitudes towards people who use drugs must also be explored further, giving attention to how effective current training is in reducing stigma in PS.

Furthermore, it is important to acknowledge the harms caused by drug law enforcement. As Fotopoulou and Aston [26] have argued, in order to safeguard public health and respect the human rights of people who use drugs, serious consideration must be given to changing the legislative context governing drugs in the UK. However, this is a slow process and as Del Pozo et al. [55] point out, the ‘public health ethic of police discretion’ may be helpful to facilitating more immediate street-level change.

While carriage and administration of naloxone by police officers has become common practice in the USA since 2014, it is still a recent development in the UK. There is still very little UK academic literature on the topic. Nor, to our knowledge, has such a detailed independent evaluation been conducted on initiatives to introduce naloxone in other UK police forces. This paper will therefore be of value to policy makers, public health professionals and other stakeholders involved in responding to rising opioid-related deaths around the world. A longer-term outcomes evaluation is necessary to assess factors such as the on-going acceptability of naloxone to police officers, rate of administration, the effectiveness of partnership working, the effectiveness of follow-up interventions and impact of naloxone and related interventions on DRDs.

The study has some limitations which we acknowledge. Firstly, there may have been selection bias among the police officers who volunteered to be interviewed or participate in focus groups. However, we spoke to officers with a broad range of views (those supportive of the pilot, those critical of the pilot and sympathetic to SPF views) and experiences (including those who had agreed to carry naloxone and those who had not, and those who had administered or witnessed naloxone administration), which provides reassurance that we captured a diverse sample. Secondly, an unavoidable environmental limitation to the research was that it took place in an atypical context, with the COVID-19 pandemic putting considerable pressure on health and police services throughout the pilot period. In addition, the UN Climate Change Conference (COP26), held in Glasgow in October 2021, put additional pressure on police resources over a period of months in the lead up to, and during, the event; a period which overlapped with the naloxone pilot. Nonetheless, this was an important study and this is the first peer-reviewed research paper reporting on carriage and administration of naloxone by police officers in the UK.

We conclude that despite tensions and concerns around the expansion of the police role, the police carriage and administration of naloxone is a valid and appropriate first aid tool and important harm reduction measure. This has been facilitated in a policy context where policing overtly acknowledges that its purpose goes beyond enforcing the law and includes enhancing the well-being and safety of individuals and communities [25]. We argue that harm reduction policing will be most effective within a public health framework, with a strong role for health and other partners, employing a whole systems approach.

## Data Availability

The datasets analysed during the current study are not publicly available as they have not been fully anonymised and may compromise participants’ right to confidentiality. Further data can be accessed in the full project report which can be found here: https://www.sipr.ac.uk/research-resources/naloxone-in-police-scotlandpilot-evaluation/.

## Abbreviations

DDTF: Drug Deaths Task Force
LEOU: Lived or living experience of opioid use
NNP: National Naloxone Programme
PIRC: Police Investigations and Review Commissioner
SAS: Scottish Ambulance Service
SPF: Scottish Police Federation
THN: Take Home Naloxone

## Footnotes

1 The training was conducted in-person and was led by police officers, supported by healthcare and legal experts. The training was presented through Powerpoint and videos. The content focused on drug-related deaths, overdose and the administration of naloxone. Details of the training are presented in Hillen et al. [28] and will be discussed further in a forthcoming paper.

2 Use of the term ‘lifestyle choice’ has been critiqued as denying ‘the extensive evidence that problem substance use is closely associated to varying degrees with poverty, adverse childhood experiences and trauma’ [31].
